# Supported in a time of need – first-time parents’ perceptions of a Swedish extended home visiting program

**DOI:** 10.1186/s12875-025-02983-y

**Published:** 2025-09-08

**Authors:** Maissa Al-Adhami, Katarina Garefalaki Kornaros, Gunilla Lönnberg

**Affiliations:** 1 Child Health and Parenting, Department of Public Health and Caring Sciences, Uppsala University, Husargatan 3, Uppsala, 752 37 Sweden; 2 Equity and Health Policy, Department of Global Public Health, Karolinska Institutet, Tomtebodavägen 18A, Stockholm, 171 65 Sweden; 3 Psychosocial work, Department of Social Work, Stockholm University, Albanovägen 18, 106 19, Stockholm, Sweden

**Keywords:** Early interventions, Home visiting program, Socioeconomic vulnerability, First-time parents, Child health services, Parental support, Access to care

## Abstract

**Background:**

Home visiting programs offer a way of delivering child health services to families that need them the most, based on socioeconomic and psychosocial conditions. Following evaluations of the implementation of a successful multiprofessional home visiting program in the Stockholm region, an extended version, *Together for a safe start*, was tested in four municipalities in the middle and southern parts of Sweden targeting first-time parents and immigrant parents having their first child in Sweden.

**Aim:**

To explore parents’ perceptions of an extended Swedish home visiting program conducted by a nurse and a social service counselor.

**Methods:**

Interviews with 17 parents (12 mothers and 5 fathers) who had participated in the program were conducted. Among the 17 interviews, 11 were conducted in Swedish, 4 in Arabic and 2 in English by a multilingual interviewer. The data were coded inductively and analyzed using thematic analysis. The findings were discussed and validated with parents from the community who advised the research team.

**Results:**

Three main themes were identified, each with two subthemes (1). Trust was created in the comfort of the home, with the subthemes ‘Home environment had many advantages’, and ‘Team’s way of working created trust’; (2) The information on child health and development was valuable, with the subthemes ‘Practical information and advice were useful’ and ‘Information on child development gave guidance’; and (3) Socioemotional support kept the parents’ worries at bay, with the subthemes ‘Mental well-being of the parents was cared for’ and ‘Parents felt supported in their new role’. Areas for improvement included mitigating the fear of social services, paying more attention to fathers’ mental health and providing more written information.

**Conclusion:**

The home environment, adaptability to parents’ needs, a combination of practical information on the child’s development, and emotional support to parents were underscored as vital components of a successful home visiting program. The results reaffirm the potential of extended home visiting programs to increase trust and make child health services accessible, as well as bridge the gap between immigrant parents and societal services.

**Supplementary Information:**

The online version contains supplementary material available at 10.1186/s12875-025-02983-y.

## Background

Home visiting programs, as a form of early childhood intervention aimed at achieving supportive conditions for children to reach their full potential, are supported by a large body of research [1–4]. The results are particularly promising for multiprofessional interventions with increased visits initiated prenatally, which target socioeconomically disadvantaged families with young children [1]. The World Health Organization (WHO) defines supportive childhood conditions as those including health, nutrition, learning and development, safety and nurturing and responsive parenting [5]. These elements are incorporated into the overall content of most early home visiting programs; however, specific goals, target populations, staffing and delivery modes can vary greatly [6]. Universal child healthcare programs, including home visits, are implemented in countries such as the United Kingdom, Germany, Denmark and Sweden [7]. In North America, programs targeting specific groups of “at-risk” factors, e.g., low income, postnatal depression and drug use are more common [8, 9].

In Sweden, the universal healthcare services directed toward parents and children under the age of two include maternal health services, child health services, and regular healthcare services. These services include access to midwives, child health nurses and physicians. More specialized professions such as psychologists, social workers, speech language pathologists, physiotherapists and dieticians that can also be solicited when needed [10, 11]. In some geographical areas, these services are provided at what is referred to as multiprofessional family centers (*Familjecentraler*), which also include open day care services. Interprofessional collaboration is encouraged in the provision of child health services [11].

The current public and child health policies that govern universal child health services and targeted services, such as home visiting programs, aim to achieve equal health in the population [11, 12]. A special focus is placed on vulnerable population groups based on the idea of universal proportionalism, which promotes the provision of universal services at a scale and intensity proportionate to the need [13]. Thus, home visiting programs and parenting support programs (outside of universal services) typically target the parents of children risking obstructed development due to socioeconomic and psychosocial vulnerabilities. Immigrant parents having their first child in Sweden have been identified as a group for whom childcare services need to be made more accessible [14]. The vulnerability of first-time parents, especially mothers, has been described in the scientific literature [15–17], justifying the prioritization of the group.

### The extended home visit program “Together for a safe start”

*Together for a safe start* is largely based on an earlier evaluated and successfully implemented home visit program (*Rinkebymodellen*) in a suburb of the capital city of Stockholm [18]. *Rinkebymodellen* started in 2013 with the aim of ameliorating the early conditions and health of children in Rinkeby, which is classified as a socioeconomically disadvantaged area. In a collaboration between child health services and social services, new to the field in Sweden [19], the number of home visits offered was increased from the regular 1–2 home visits usually occurring at one and eight months, to six visits to ensure that the first-time parents that were the target group would receive more information and more support early in their children’s lives. Following evaluations showing promising outcomes [19–22], the program became standard practice in 2017 and was implemented in other areas in the Stockholm region.

*Together for a safe start* used the same methodology as *Rinkebymodellen*, but the original six home visits were complemented with two extra visits: one maternal clinic visit at pregnancy week 34 and one visit at the child healthcare clinic at 24 months, led by a child health nurse and a parental counselor from social services. Commissioned by the Swedish government through the National Board of Health and Welfare, the aim was to strengthen the conditions for satisfactory and equal health among children. To fulfill the aim of equal health, the program focused on first-time parents, and immigrant parents having their first child in Sweden, living in socioeconomically disadvantaged areas where the need for parental support is hypothesized to be higher due to intersecting vulnerabilities. Four areas in four municipalities were included in the program. The areas were selected on the basis of two main criteria: (1) the Swedish Care Need Index (CNI), which identifies risk for ill health based on seven socioeconomic variables, such as employment rate, educational attainment, number of foreign-born and single parents in an area [23], and (2) classification of vulnerable areas by the Swedish Police Authority. The municipalities also had to agree to be part of the program. In total, the extended home visiting program reached 87% of the eligible families in the four target areas (186 out of 213 families).

The program started with an introductory session taking place at the maternal health clinic or a family center for some sites in late pregnancy (around gestational week 34). The first home visit occurred when the child was newborn, i.e., 1–2 weeks old. This was followed by home visits at 1–2, 4, 8, 10, and 15 months of age. The program ended with a visit at the child healthcare clinic when the child turned two years old. Altogether, the program offered six home visits and two visits at the clinic (see Table 1). In addition, regular visits to the childcare clinic included in the Swedish child healthcare scheme were scheduled, encompassing eight clinic visits for the purpose of vaccination and physical examination including measuring the child’s weight and length [11]. At the home visits, a multiprofessional team consisting of a child healthcare nurse and a parental advisor or a social counselor from the social services were present. Specific themes were presented to and discussed with the parents at each visit following a guideline [24]. Questions were encouraged, as was flexibility to incorporate other topics suggested by the parents. The timing and core content of the visits (i.e., type of information provided and discussed by the multiprofessional team) are summarized below (Table 1).

**Table 1:** Content and timing of the visits in the program

Type of visit	Time	Theme
Prenatal visit at clinic*	Pregnancy week 34–38	**Becoming a parent & introduction to the program** - Comprehensive information about the program - Being a parent - The first period at home with the baby - The family and other significant adults
Home visit 1	Week 1–2	**Receiving your child** - Food (breast-feeding, infant formula) - Child’s safety (e.g., sleeping on their back) - Engaging with the child (talking to, being near and comforting the child) - The parents (being a team, psychological impact of becoming a parent, social networks & support)
Home visit 2	Months 1–2	**Getting to know your child** - Child’s temperament, personality and signals - Communicating with the child (talking, playing, and reading to the child) - Regulating emotions and giving time - Parents’ communication and helping each other - Colds, fevers and caring for a sick child - Activities (parental groups, open day care services)
Home visit 3	At 4 months	**Being together** - Stimulation and development (play, language development) - Interaction (parent/child) - Planning of parental leave - Food and sleep routines - Activities for children and parents
Home visit 4	At 8 months	**Leading and following** - Stimulation and development (the importance of playing together) - Food, sleep and teeth routines, potty training - Managing the child’s curiosity and will - Guiding the child (diversion and attention) - Safety - Nursery school
Home visit 5	At 12 months	**Creating a daily life** - Food and meal routines (e.g., healthy food) - The importance of play and exercise - Guiding the child and setting boundaries - Handling the child being more active - Infections and caring for the sick child
Home visit 6	At 15 months	**Being a family** - Language development (words, comprehension, other ways of communication) - Nursery school routines and planning - Continuing to play together - Cooperation between parents - Other significant adults
The 2-year visit at clinic**	At 24 months	**Growing and developing together** - Spending time together as a family - Language development - Food routines and dental health - Play and exercise - Nursery school – spending time with others - Thoughts about the future

* Maternal health clinic

** Child healthcare clinic

The aim of this study was to explore the perceptions of first-time parents – including immigrant parents having their first child in Sweden – of an extended home visiting program led by a child health nurse and a social service counselor.

### Theoretical framework

Access to care is a vital marker of the performance of healthcare systems globally. When initiating a new or modified type of healthcare service, the access component must be explored from both health system and provider perspectives as well as from community and individual perspectives. Access to care has been conceptualized by Levesque to include five dimensions that can be used to measure the usefulness of a given healthcare service: approachability, acceptability, availability and accommodation, affordability and appropriateness [25]. In the framework developed by Levesque et al., five corresponding abilities of the user or population to interact with these dimensions help generate access: ability to perceive, ability to seek, ability to reach, ability to pay, and ability to engage. By employing an access lens, the healthcare needs, reach and potential of the service can be mapped out. For the purpose of this study, we focus on the aforementioned five dimensions of the supply-side aspects of the framework. *Approachability* refers to people with health needs being able to identify that services exist, obtain information about them and actually reach them. *Acceptability* is related to factors that determine acceptance of the service, e.g., the professional values and norms of healthcare providers. Linked to this is the *appropriateness* dimension, which explores the fit between services and needs, their timeliness and the technical and interpersonal quality of the provided services. *Availability and accommodation* refer to services being physically and otherwise available (locations, opening hours, appointments, etc.). Affordability denotes direct and indirect costs affecting the possibility of utilizing healthcare services. We employ these concepts to discuss the results of the study.

## Methods

### Setting

The *Together for a safe start* program was initiated in 2020 and started in 2021 in four municipalities in the middle and southern parts of Sweden. The evaluation study started in 2023. Following the overall goal of the program, the program was centered on four socioeconomically disadvantaged areas in the respective municipality. Växjö, Linköping, Eskilstuna and Borlänge are medium size municipalities (with a population of 40,000–200,000) [26]. The study was carried out in four areas Araby (Växjö), Skäggetorp (Linköping), Fröslunda (Eskilstuna) and Jakobsgårdarna (Borlänge), categorized as vulnerable areas by the Swedish police authorities, ranging from ”at risk” to “very vulnerable areas”. The post-secondary education levels in these areas were lower than the Swedish national average, ranging from 11 to 28% compared to 44% for the general population. The proportion of residents with employments was also lower in all areas, compared to the Swedish national level, ranging from 50 to 67%, compared to the national level of 80%. Moreover, the proportion of residents born outside of Sweden was larger than the Swedish average in all four areas, 50–72% compared to the national level of 26% [27].

### Participants and recruitment

The inclusion criteria were being a first-time parent or having had a child for the first time in Sweden and having participated in the program. Nurses at the child healthcare clinic asked one group of parents who were eligible if they wanted to participate in the study. Upon agreeing to have their contact information shared, they were contacted by the research team. Another group of parents who had participated in a quantitative evaluation of the effects of the program on health outcomes were contacted directly by the research team and asked if they wanted to participate. The recruitment continued for several months (in parallel with the data collection), as it was difficult to reach parents. This was due to some having moved from the area, some clinics not reaching or asking all eligible parents, and some parents declining to participate. In total, 20 parents were reached. Three of the contacted parents opted out of participation (15%) and 17 parents consented to participate and were interviewed. Of these, twelve were mothers, and five were fathers. All were first-time parents except one mother, who had immigrated to Sweden and had her first child in the country (and one older child born outside of Sweden). The demographic characteristics of the participants are shown in Table 2.

**Table 2:** General characteristics of the participants (*n* = 17)

Gender	Number
Female (mothers)	12
Male (fathers)	5
Age	
20-30	8
30-40	6
40-50	3
Geographic area	
Växjö	6
Linköping	4
Eskilstuna	4
Borlänge	3
Country of birth	
Sweden	8
Somalia	3
Syria	2
Yemen	1
Sudan	1
India	1
Iran	1
Language (in which interview was conducted)	
Swedish	11
Arabic	4
English	2

### Data collection

An interview guide with one opening question and nine main questions with probing questions about the program was developed. The interview guide was translated into English and Arabic and back translated to validate accuracy and quality. At the beginning of the data collection, several modes of interviewing, i.e., telephone, video conference and face-to-face, were offered to the participants. All participants in the initial group opted for telephone interviews attributing it to being more practical for them, which led to the decision to conduct the remaining interviews by phone. Each interview lasted for 25–50 min and was recorded with permission from the participants. The interviews were carried out in three languages (Swedish, English and Arabic) by the first author, who is multilingual. The interviews that were conducted in English or Arabic were translated and transcribed into Swedish by a multilingual research assistant working for the research team. The translation/transcription was checked by the first author. All the recorded interviews were transcribed verbatim. The data collection was carried out between June and October 2023.

We determined data saturation when finding that the data in last interviews repeated what was expressed in previous interviews, rather than providing new information or insights, referred to as data collection saturation [28].

### Community advisory group

To ensure that the perspectives of the community was included in the study, the research team recruited a group of (non-participant) mothers, to act as an advisory group to the researchers (hereinafter referred to as community advisors). The community advisors were recruited via social media channels and local associations in one of the target areas of the program. The group consisted of 11 immigrant mothers, who had given birth to, or were raising children in the target area. Their role was primarily to provide feedback on the analysis of the data (see Analysis section below), specifically on findings relating to immigrant parents who were a majority of the participants. Their participation constituted a public involvement dimension, which is suggested to increase communities’ interest and agency, as well as legitimacy and credibility to community intervention studies [29, 30].

### Researcher reflexivity

The research team consisted of three female researchers (Ph.D.) and one female research assistant in charge of the project administration. Two of the researchers have a public health background, and one has a background in psychology and sociology. All three worked as researchers and teachers in academic institutions in Sweden at the time of the study. In combination, the research team has extensive experience working with intervention programs involving parents and parenting, health promotion and health literacy, immigrant and vulnerable populations, and qualitative methodologies. Two of the researchers are Swedish-born, and the third (first author) has an immigrant background with language skills that are pertinent to the study. As authors, we position ourselves in a realist/social constructivist tradition that recognizes the role of social interaction and culture in shaping people’s constructions of meaning, knowledge and reality. A starting point for our research is the frameworks of social determinants of health and health equity. As researchers within the field of public health and health sciences, we acknowledge that our understanding and perceptions of the research topic could carry implicit biases (such as focusing on certain patterns in the data). To counter this, we opted to include non-researchers, i.e., the community advisors to give input during the analysis phase as described below and in Table 1.

### Ethical considerations

Before the start of the project, an ethical application outlining the purpose and process of the study, including recruitment, data handling and data storage, was developed. Permission to conduct the study was granted by the Swedish Ethical Board in Gothenburg (reference number 2022-01493-02). As part of ethical considerations, written information about the study was developed in several languages, i.e., English, Arabic, and Somali. The parents who were recruited also received oral information about the study by nurses at the child healthcare clinic as well as by the research team. The written information included the aim and procedure of the study, handling and storing of data, and contact information with the principal investigator. It also stated that participation was voluntary and could be discontinued at any time during the study. We obtained oral informed consent from all participants ahead of the interviews as well as permission to record the interview session. To ensure the anonymity of the participants, all personal data were removed from the quotations in the Results section.

### Analysis

**Fig. 1:**
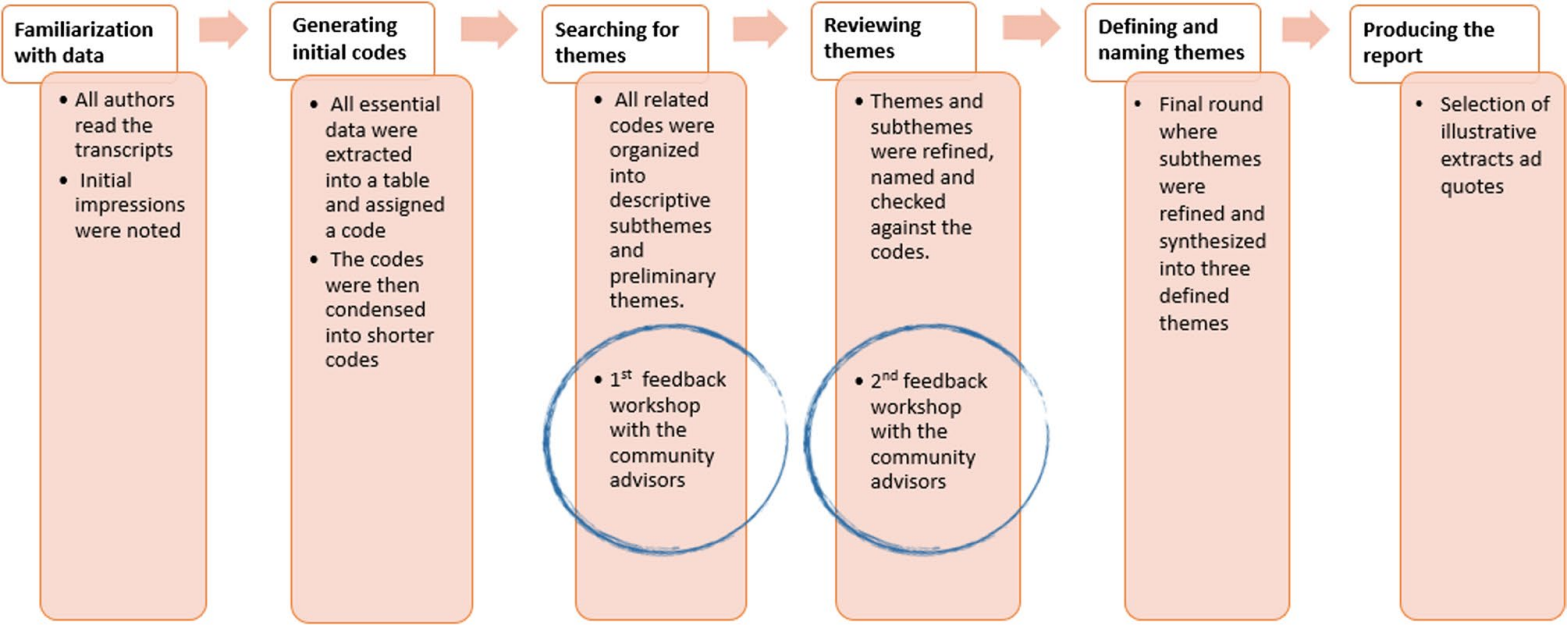
Schematic steps in the analysis process, including feedback loops

The data were analyzed inductively following the steps of thematic analysis described by Braun & Clark [31] as illustrated in Fig. 1. As a first step, all the authors familiarized themselves with the data by reading the transcripts. Initial ideas and impressions of the dataset were noted by the authors independently and compared in a first meeting. This step allowed for a common understanding of main features of the data and helped guide the next round of coding, where all transcripts were coded more thoroughly by the first author and one transcript by the second author for validation and conformation of main codes. The coding process included three steps: all essential matters discussed in the transcripts, usually spanning a sentence or two, were extracted into a table (MS Word) and assigned codes; the codes was then condensed into shorter codes; all related codes were then organized into descriptive subthemes and preliminary themes. Variations in the dataset were noted and included. The themes and subthemes were discussed and checked with the community advisors recruited by the research group to increase legitimacy and credibility to the interpretation of the data [32]. Their role was to provide feedback on the emerging themes and sub-themes that were presented to them in two separate workshops, specifically findings relating to immigrant parents who consisted a majority of the participants. A second analysis round involving all three authors was then performed where the themes and subthemes were refined, named and checked against the codes. This analysis was then presented a second time to the same community advisors, who gave input and validated the themes. After this, the authors met for a third and final round in which the subthemes were refined and synthesized into three defined final themes. Quotes from the respondents are used to highlight the findings.

## Results

**Fig. 2:**
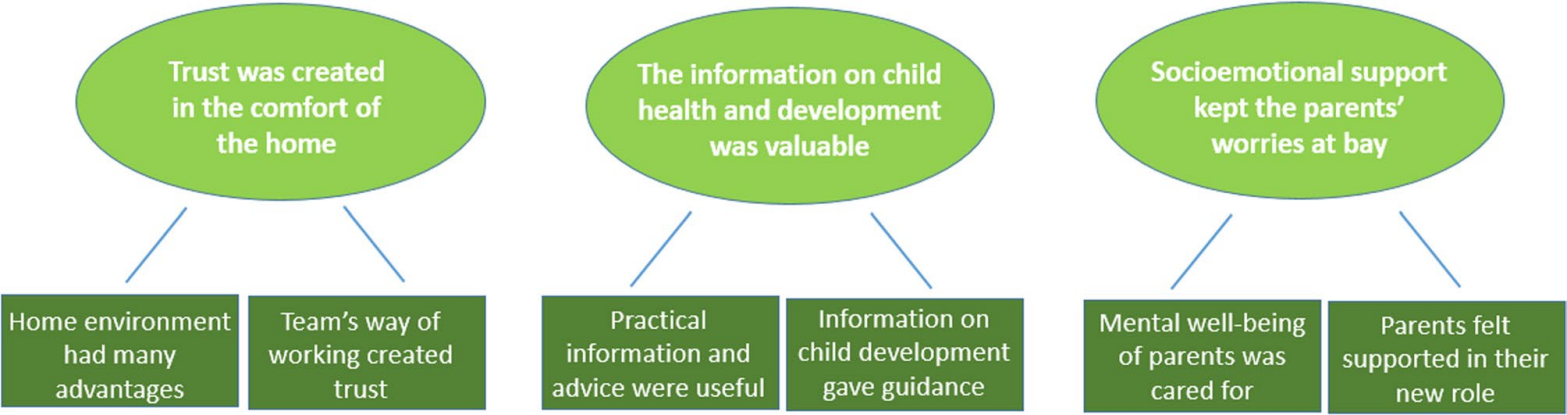
Thematic map – last sequence of analysis

Three main themes were derived from the data: (1) Trust was created in the comfort of home, (2) The information on child health and development was valuable, and (3) Socioemotional support kept the parents’ worries at bay. Each theme was synthesized from subthemes that are presented below and illustrated in Fig. 2.

### Trust was created in the comfort of the home

The theme Trust was created in the comfort of the home was developed from the subthemes ‘Home environment had many advantages’ and ‘Team’s way of working created trust’.

#### Home environment has many advantages

A majority of the parents talked about home visits as an advantage from a practical and logistical point of view; obtaining access to child health nurses and parental counselors without having to leave the house was described as both time and cost saving. All the respondents regarded visits to the childcare clinic as a necessary complement; however, not having to travel to the clinic or family center with a small child in wintertime or bad weather was mentioned as a benefit of the program. More prominently, the home environment as such was highlighted by several parents as having many advantages, not least allowing for greater help. Moreover, a few parents mentioned that home visits enabled the child health nurse and parental counselor to give more time to the parents and children and that they were less stressed for time than clinic visits.

*“They took it on our terms. They didn’t come to our home*,* were stressed*,* and just wanted it (the visit) to be over with. We sat and talked and it took the time it needed too*,* and that felt very good.*

*(Father, #1).*

A majority of the respondents mentioned the possibility of seeing the child and parents in their natural environment more than once and receiving feedback on issues related to both practical and developmental issues as an advantage. The child health nurse and parental counselor could, for instance, observe the child and the parent‒child interaction in a familiar setting described as one where the child could be more at ease:

*“If you have shy children*,* then you can see when they (the multiprofessional team) come to your home that*,* yes*,* they can speak*,* they can move and walk and so. Many children hide a bit (in the clinic) and may not show anything until you come home.” (Mother*,* #2)*.

Another advantage mentioned by the parents was that the team could see and provide advice about how to make the home environment safe and fit for the child, e.g., the sleeping place, kitchen and playing areas. The safety of the home was an issue that many parents described as one that they appreciated receiving information about:

*“And then, when they come to your home, well, I could even show the bed and ask, but is it safe? Should it be like this? It was at the time when she (the baby) had started to stand up and hold on to the cot, and I was unsure if she could climb over it and fall. It’s not easy to explain something like that when you are at the clinic.” (Mother*,* #3)*.

#### Team’s way of working created trust

Several parents mentioned the multiprofessional team’s expertise in the area of child health as well as their approach and the methods that they employed, which was especially appreciated. The theoretical information provided by the team on, for example, language development and play was coupled with practical guidance *how* to speak and *how* to play with the child in the home environment. Some parents reported that they remembered what was shown to them in their home more than what was shown in the clinic:

*So they showed in our home (leg and belly massage) because I felt that when you were there (at the clinic) and got tips about these things…but then when you left the room*,* you forget it quickly and had to sit and think about it. But here*,* you were in your home*,* and you could pin it to your memory in another way. ” (Mother*,* #2)*.

The respondents reported having between 4 and 8 mandated visits within the program, and some reported more visits. The prenatal visit at the maternal health clinic was identified as important; some who had not had this visit pointed out that it would have been helpful to know beforehand what type of services they would receive. Having the same child health nurse and parental counselor through the duration of the program and an adapted number of visits on the basis of need was mentioned as particularly important by some parents. This was mentioned in relation to having specific circumstances warranting extra support, e.g., having had a difficult delivery, illness of a parent or child, being new in Sweden or parenting alone.

One mother commented that home visits with parental counselors from social services could be a challenge. She mentioned that others in her community expressed concern that they were there to control the parents. This was corroborated by a father who commented that he had been nervous about receiving the childcare nurse and parental advisor at first but that their dedication had won him over:

*“It became easier*,* we got to know them*,* and it became easier. We knew what they were going to talk about and that they were there to help us […] They made us feel at ease*,* if you know what I mean? They didn’t make us feel that they were there to give us advice.” (Father*,* #4)*.

A majority of the parents reported that they were informed at the start of the visit what themes were going to be brought up. The information sheets with the themes were available in different languages. The dialog and possibilities to ask questions outside of the fixed themes for each visit were mentioned as especially valuable. Most parents reported that they felt that there was a good balance between the predetermined structure of the home visits and an adaptation to the needs of the parents. If they wished to speak about a certain subject such as the infant’s sleep, the team would make room for it. The child health nurse and parental counselor were perceived as a team with an approach that created trust:

*“We have gained a bigger trust in (them); it feels like we have better contact with our child health nurse. As I said*,* it could be a bit formal if you just meet at the clinic; at home*,* it’s more familiar. You sit and play with the child on the floor while talking*,* and that conversation becomes more natural.” (Father*,* #5)*.

### The information on child health and development was valuable

This theme was developed from the subthemes ‘Practical information and advice were useful’ and ‘Information on child development gave guidance’.

#### Practical information and advice were useful

The respondents perceived the practical information that was included in the program as useful and relevant. The topics related to the child’s physical health that were mentioned most frequently were food, sleep, safety, play and spending time outdoors. Some respondents also reported having received information about preschool and parental education. For some respondents, the information was new, whereas others reported that they knew or had acquired knowledge about some of these topics on their own, for instance, through internet resources or families and friends. Even if that was the case, some foreign-born parents shared that they wanted to learn more about Swedish child health services’ recommendations on these topics. Some single parents and parents new to Sweden also reported that they received useful information about public and municipal services suitable for children and parents, e.g., open daycare centers, recreational services, and libraries.

Moreover, in addition to the information that was part of the fixed themes of the program, all parents appreciated being able to ask questions and receiving advice on a specific question or problems related to their child’s health and development, e.g., the child not eating or not sleeping for longer periods of time:

*"Yes, after three months, we were really tired and then we told them we are not getting any sleep, please help us, and then they told us how to convert the child from sleeping with us to the crib. They helped us, they assisted us, they told us how to do it, and we tried it, we also did it." (Mother, #6).*

#### Information on child development gave guidance

A majority of the parents expressed an interest in information related to the child’s cognitive and social development. They mentioned that it was good to learn at what age a child was expected to talk or walk and what the parents could do at different stages to promote their child’s development. They perceived the topics that included language development and how to interact, talk to and play with the child as important. Furthermore, they included practical tips and advice that the parents could use and follow. Multilingual parents shared that they were encouraged to talk in their mother tongue with the child. Several parents shared that the information about reading books to the child early on was useful:

*“We got information about books and that you should read books to children when they are small. They understand even if they are at a young age. And it makes it easier for them to start talking when they are small”. (Father*,* #7)*

Some parents expressed that learning how to talk with the child, e.g., and describe what the child was doing, as well as putting words to their feelings, was valuable. The role of play was also mentioned as beneficial and especially appreciated when it was coupled with practical guidance:

*“I don’t know how to play with my daughter; I mean*,* I sometimes feel that I want to play with her*,* but I don’t know how. This isn’t something that I’ve learned when I was growing up*,* and she (the nurse/parental advisor) showed me*,* you know what to do*,* what to do with her and that I should talk to her.” (Mother*,* #3)*.

Two parents to twins related that most of the practical information on child health and development was obtained for single children and was not adapted enough for twin parents. One parent also expressed that she was referred to internet resources for certain information instead of being provided with it. Some parents received brochures and written information about some topics, such as food and recipes for infants, whereas others wished for written information about other topics, such as the child’s development. A majority understood the role of the child health nurse as the one providing practical information and the parental advisor being more in charge of the developmental part. Nevertheless, for some respondents, this distinction was less accentuated; the person and personal rapport established was described as equally important as the function.

### Socioemotional support kept the parents’ worries at bay

This theme is synthesized from two subthemes emerging from the data: ‘Mental well-being of the parents was cared for’ and ‘Parents felt supported in their new role’. The two sub-themes will be presented in conjunction as they were interwoven in the way that participants discussed them.

The topic of the mental well-being of the parents was discussed by all the participants. The mental health of parents was viewed as multifaceted. It was related to the life transition of becoming a parent, worries related to both the child’s development and the parent’s ability to be a good parent as well as being new to the country and other vulnerabilities, such as being a single parent or suffering from postpartum depression. Becoming a first-time parent was described in itself as constituting a new vulnerability:

*“It’s the first time you are responsible for another human being’s life. You feel responsible*,* and it is truly hard; it’s a special feeling.” (Father*, #7).

All parents expressed the joy of interacting with their newborn babies and that becoming a parent was life changing in a positive way. The parents expressed emotions such as happiness, giving and receiving unconditional love, joy and contentment in life. However, they also saw a need to normalize talking about mental health and that it was okay to feel sad as well as happy. Here, a few participants noted that it was good that the multiprofessional team asked about the parents’ mental state:

"*[…] Even about my well-being, I remember that the nurse asked me questions. She asked me after the delivery about depression and my feelings and my relationship with the baby." Mother, #8).*

In addition to starting the visits by asking about the well-being of the parents, a few participants shared that the parental counselor also informed them about the mental health counseling that was available. A few parents noted that it took time for them to start talking about their mental health and that they gave more general answers in the beginning and disclosed more after having established a relationship with the team. Two fathers noted that questions about mental health were often directed to mothers. While understanding that from, e.g., the perspective of postpartum depression, they felt that fathers’ mental health should be further addressed and supported. They described how they, as fathers, struggled to be useful in the first six months when the babies were naturally more attached to the mothers due to breastfeeding.

*“The hard part was that I couldn’t help out with the child. Because it was only being with the mother that did the trick. It became a negative trend*,* and I would feel rather worthless because you cannot help*,* as you want to. And it wasn’t until I got the ”father-talk” (session dedicated to the fathers)*,* three or four months after I could start expressing that feeling […] You notice when you talk to other fathers that they were also not feeling good*,* that you are not prioritized (in the program) and that there isn’t much talk about it. It’s hard on fathers too*,* psychologically that is”.* (Father, #5)

Correspondingly, a few mothers expressed that being solely responsible for feeding the infant (breastfeeding) and having to take the larger part of the infants’ other routines during the first six months was both physically and mentally draining. Feeling supported as a parent was equally important to all participants regardless of prior knowledge about different aspects of the child’s development. To be supported in the first six months of the child’s life was mentioned as particularly important by almost all parents, as first-time parents struggled with establishing routines for their infants:

*"I remember the first visit, I was desperate, you know…the breast-feeding, and I wasn’t feeling good, and then they came and truly saw me…received my questions and answered them. It felt like I had people who really cared, and I got their telephone number too so that I could send a message if something came up. I just felt that I had such a trust for them. That they were there to help me."(Mother, #3).*

A majority of parents described being worried about different aspects of their children’s development, such as their social abilities, motor abilities and language development. They also had catastrophizing thoughts of disasters, such as sudden infant death syndrome and the child choking on food. The multiprofessional care team was described as being good at explaining and giving facts about these issues, often leading to parents feeling less worried. Two parents mentioned that parental support groups and courses were cancelled due to the COVID-19 pandemic, which led to more isolation and increased worry and dependence on home visits. A single father who had recently moved to Sweden explained that the added visits made him feel less lonely.

Another concern for first-time parents was the child starting on day care, a concern that was also addressed by the childcare nurse and parental advisor. Single parents and other parents with specific vulnerable situations (such as one where the mother had postpartum depression) often expressed socioemotional support as one of the most important aspects of the home visiting program. The ability of the multiprofessional team to connect with them and support them in their parenthood could be expressed as follows:

*“We are friends; I say to my child*,* you have another family*,* the childcare services. Because they help me a lot. I live alone*,* I don’t have family here […] The childcare services were my family. I know if I needed something to ask them.” (Father*,* #9*).

## Discussion

The results consisted of three themes: trust was created in the comfort of home, the information on child health and development was valuable, and socioemotional support kept worries at bay. The program was viewed as providing a balance of practical information about the child’s development and emotional support to parents. The home environment stands out as an important factor for creating trust between the multiprofessional care team and the parents. Being a first-time parent to an infant is described not only as having positive emotions but also as being fraught with vulnerability. Inexperience, isolation and worry are mentioned as being taxing on mental health. From the parents’ perspective, the extended home visiting program addressed many of these issues. The program content, the multiprofessional teams’ ways of working and their flexibility were viewed as success factors. Areas that could be developed further from the point of view of the respondents were information specific to twin parents, more attention to fathers’ mental health and more written information. To have the same staff on the multidisciplinary team and the same interpreter (when those services were utilized) were mentioned as important by both parents and community advisors. The latter stressed the potential of the home visiting program to increase trust, decrease isolation and bridge the gap between immigrant parents and other societal services.

Overall, our results confirm the benefits of an extended home visiting program found by other studies, specifically in relation to socio-economically vulnerable families with children [1]. The study offers additional insights on issues related to immigrant parents’ perspectives on social services participating in the home visits and fathers’ perspectives on mental health needs. The study also highlights primary healthcare access aspects within the scope of home visiting programs.

### The access dimensions of the Levesque model

In the Levesque model (see Theoretical Framework), the provider side of access to healthcare is understood to be multidimensional [25], centered around five main areas: approachability, acceptability, availability, affordability and appropriateness. We will discuss the results in relation to these dimensions.

#### Approachability

In our results, the parents regarded the first prenatal visit as essential, a visit initiated by the maternal health care services. The information they received by the midwives provided clarity about the type and content of services offered through the extended home visiting program and gave parents more time to prepare for what they had consented to. This is consistent with the approachability aspects transparency and information about the service being adequate for the parents who received the information beforehand, but less so for the parents who received the information at the first postnatal visit. A review study by Entsieh et al. [33] found that new parents needed prenatal information on parenting and where to find support from health professionals in the early phases of the parenthood. The importance of being informed ahead of time was also stressed, in line with our findings.

With respect to the information provided by a midwife (and not the team conducting the home visits), Bäckström et al. [34] reported that parents participating in similar home visiting programs perceived midwives working in prenatal care as a cohesive part of the childcare services team. Burström et al. [20] point out the importance of the program reaching parents in socioeconomically disadvantaged areas, where the need for services is understood to be greater. The parents participating in the program, living in such areas were approached by regular healthcare services who thereby increased the reach of the services, which is an essential part of the approachability dimension. Moreover, a Danish study by Hirani et al. [35] showed that home visits *ahead* of the first vaccination, increased the likelihood of timely vaccination, especially for first-time parents, adding to the approachability dimension.

#### Acceptability and appropriateness

The willingness of the staff to adapt to the parents’ situation and their readiness to provide psychosocial support were mentioned by several participants. This could be in the form of added visits, altered information, e.g., more information about societal services to foreign-born parents, and more support to single parents. Their technical abilities, e.g., conveying relevant practical information in a clear way, answering parents’ questions, and showing parents how to play and interact with the child, were equally highlighted. These findings are consistent with the *acceptability* and *appropriateness* dimensions being sufficient, denoting aspects such as professional values and technical and interpersonal qualities. A recent mixed-method study by Lundgren et al. found that competence, ability and engagement facilitated the implementation of a home visit program [36], in line with the parents’ views in our study. The expressed gain of information and support is consistent with results from other studies exploring parents’ views of home visiting programs, especially those involving immigrant parents [22, 37]. Tittinen et al. found that fathers who had participated in an extended home visit program gained increased resilience and acquired new knowledge of services and resources available to them [22]. However, a recent Norwegian study [38] found that an increased support and attention to fathers’ needs and emotions is warranted, which is echoed by our findings. Moreover, the introduction of the parental advisor into the care team for the home visiting program has been shown to add value to the program from the perspective of parents and enable a wider scope of information and services [19]. Simultaneously, a multidisciplinary team requires collaboration skills and the same staff (and interpreters) to achieve continuity and greater acceptability. The appropriate timing of the program was corroborated by the parents. Firstly, that the program targeted first-time parents and immigrant parents who had their first child in Sweden, and secondly that it began at the start of the child’s life, a time, when the need for support was perceived as greater. The timeliness of the home visiting program has been highlighted as an important factor for success by other studies [20].

#### Availability and accommodation

The home visiting feature of the program increased the *availability and accommodation* of the program, referring to the physical and logistic aspects of the delivery of healthcare as well as characteristics of the healthcare provider. Parents expressed how home visits coupled with visits to the clinic made logistics easier and helped them accommodate their needs as first-time parents. Several examples are given in our results concerning how the home environment made both parents and children more at ease, enabling more natural and practical interaction with the childcare nurse and parental advisor. This finding is well established in research on home visiting programs [22, 34]. Importantly, our findings suggest that trust is created in the comfort of the home. Trust is linked to characteristics of the healthcare provider, such as ability, skill, benevolence and integrity [39], in turn linked to the acceptability and appropriateness dimensions of the offered care. An illustrative example of this is that some parents were reluctant at first to receive the parental advisor being part of the social services, in their home. The advisors were however able to build a trusting relationship that made the parents feel at ease and actively engage with them and the program. Larson et al., reported that healthcare staff regarded home visits as an opportunity to build a lasting relationship with parents and gain new insights [40]. This finding is in line with what the community advisors expressed, explaining that the social services coming into homes was an opportunity for the parents to get to know the social services but also for social services to get to know and learn from the parents. They saw this as a way of building a lasting trust that would enable parents to reach out to social services later in the child’s life if they needed their help. Similarly, a Norwegian study by Holmberg et al. on the perceptions of nurses of a home visiting program [41] report that the program was seen as valuable for building relationships and trust. The concept of trust is central to access to healthcare and has been suggested as a lens through which welfare programs such as the current can be studied [42]. Our results suggest that trust created within the process of the home visiting program may translate into greater trust in child healthcare services and a greater willingness to engage with social services in later stages of the child’s juvenile and adolescent years. This has been echoed by other studies, stressing the gains for immigrant parents in particular [22, 34].

#### Affordability

With respect to the issue of affordability, child healthcare services in Sweden are free of charge, which eliminates direct costs. For indirect costs, some of these costs, such as travel expenses, are lower in the case of the home visiting program, which thus results in higher affordability than do clinic visits. The time- and money-saving aspects for parents were mentioned in the results, indicating that the issue is not of minor importance.

### Strengths and limitations

The data were collected via telephone interviews, which might lead to less interaction than face-to-face interviews. On the other hand, the telephone was the preferred way of being interviewed by parents, described as being more practical and timesaving in a demanding phase of their parenthood. An illustration of this was that some parents partook in the interview while being at home with the child. Additionally, research suggests that telephones may allow respondents to feel more at ease and be able to talk more freely, but evidence that the data are lower in quality is lacking [43].

As in most empirical studies, there is a risk of selection bias, where parents who perceive the program as useful and positive opt to participate to a greater extent than those who are dissatisfied. However, the demographic composition of the participants varied, enabling more perspectives to be brought up, and aspects that were lacking in the program were conveyed. Additionally, some participants had previously participated in a quantitative evaluation of the program. Although different in nature, and performed by different researchers (results were not disclosed to us), this might have influenced the participants’ decision to partake in the interviews and possibly affected their answers, although it is difficult to determine in what ways.

A strength of the study was that the Swedish-, English- and Arabic-speaking parents who participated in the interviews could be interviewed in their mother tongue by the same interviewer (first author), which enabled a richer conversation, avoiding loss in information due to interpretation [44].

### Practical implications

- An extended number of home visits within the first two years of the child’s life benefit parents in providing them with information and guidance on their child’s health and development, and in making child healthcare and social services more accessible.
- Having the same child healthcare nurse and parental counselor throughout the duration of the program, and the number of visits adapted to the needs of the family, is beneficial from the point of view of parents.
- Having a focus on first-time parents’ socioemotional needs and providing support is an important part of the home visiting program.

Longitudinal studies comparing effects on developmental and health outcomes in parents and children are warranted to understand the long-term effects of extended home visiting programs.

## Conclusion

This study adds knowledge on benefits and challenges in an extended home visiting program led by a multiprofessional team from the perspectives of first-time parents. In particular, immigrant parents’ and fathers’ perspectives are elucidated, as well as access dimensions of extended home visit programs as part of the primary healthcare system. The results point to the home environment and having the same staff and the same interpreter (when utilized) as important for increasing trust, decreasing isolation, and bridging the gap between immigrant parents and other societal services. The program’s adaptability to parents’ needs and the combination of practical information on child health and development and emotional support emerged as other vital components of a successful home visiting program. Areas for improvement included mitigating the fear of social services, paying more attention to fathers’ mental health and providing more written information. The trust created between the multiprofessional care team and the parents could translate into a greater willingness to solicit child healthcare and social services later in the child’s life. The combined results reaffirm the contributions of extended home visiting programs in making primary child healthcare accessible. 

## Supplementary Information

Supplementary Material 1.

## Data Availability

The data analyzed during the current study are available from the corresponding author upon reasonable request. To protect the participants’ identities, the full interview data (audio files and transcripts) will not be made available to the public.

## Abbreviations

WHO: World Health Organization
CNI: Swedish Care Need Index
