# Supplementary material for: Supported in a time of need – first-time parents’ perceptions of a Swedish extended home visiting program
Source: BMC Prim Care. 2025 Sep 8;26:281. doi: 10.1186/s12875-025-02983-y (PMC12418617; doi:10.1186/s12875-025-02983-y)
Supplement: Supplementary file 1 — Supplementary Material 1. [file 12875_2025_2983_MOESM1_ESM.pdf]

Interview guide - Parents who participated in *Together for a Safe Start*

To start with, can you tell us how old you are and if this is your first child or if you have more? How long have you lived in (*name of area, in Sweden if foreign-born and country of birth*)?

What has this first time with your child been like for you? What have the home visits meant to your family?

Ask to elaborate and give examples. Topics:

- *Confidence in their parenting skills*
- *Interacting and playing with the child, movement, outdoor activities*
- *Child development, especially language development*
- *Food, sleep and dental health routines*
- *About siblings - allocating attention, boundary setting*
- *Trust in society's resources*
- *Social network and support*

Has the child's other parent *(mother/father/co-parent)* also been involved in the home visits? How has it been for you with participation and equal parenting? Expectations of each other in parenting?

This last visit, when your child was two years old, can you tell us about what it was like? Was there anything you talked about during the visit that you found interesting or helpful?

Was there anything raised by the staff that you found unhelpful or uninteresting? *(If so, please tell us what)*

Is there anything you wish you had talked about that you did not? *(Can you tell us more about this...)*

Do you have any suggestions for improving this program from the start during pregnancy until now?

Is there anything else you can think of, anything you would like to add?
